# Band Gaps and Transmission Characteristics Analysis on a Two-Dimensional Multiple-Scatter Phononic Crystal Structure

**DOI:** 10.3390/ma13092106

**Published:** 2020-05-02

**Authors:** Hang Xiang, Xingfu Ma, Jiawei Xiang

**Affiliations:** College of Mechanical and Electrical Engineering, Wenzhou University, Wenzhou 325035, China; hsiwyvern1024@163.com (H.X.); 184511482352@stu.wzu.edu.cn (X.M.)

**Keywords:** multiple-scatter PC structure, finite element method, band gaps, starting frequency, transmission characteristics

## Abstract

In this paper, a novel wrap-around multi-scattering phononic crystal (PC) structure is proposed. Band gaps (BGs) and transmission characteristics of the present structure are calculated using finite element method (FEM). Through the calculations of single-scattering prototype, three complete BGs which are exhibited at low frequency and the fourth wide BG at high frequency are discovered. The transmission features and resonant spectra represented by frequency response function (FRF) shows that apparent resonance directly cause the four specific BGs. By keeping the total area of scatterers unchanged, 2 × 2, 3 × 3 and 4 × 4 scatterers are designed to obtain the change rule of BGs. Furthermore, the size ratio of 2 × 2 scatterers, the number of connection beams are investigated to obtain the regular pattern of acoustic energy transmission and attenuation. The present investigation of multiple-scatter PC structure will provide a solid support on the future design of acoustical functional materials.

## 1. Introduction

To the demand of noise and vibration reduction in daily life and industrial world, the concept of phononic crystal (PC) has been proposed and explored [1,2]. After that, scholars focused on the development of composite PCs (PCs are always periodic) and laid emphasis on the theoretical and experimental research of elastic wave propagation [3,4,5,6,7,8]. The extraordinary acoustic properties and physical characteristics of PC structures show a wide application prospects in noise cancellation, vibration suppression, acoustical filters and wave guides, etc. [9,10,11]. Therefore, PC structures have drawn attention with the plenty of enough preponderances. The existence of band gaps (BGs) is one of the momentous characteristics of PC structures. Based on the generation mechanism of BGs, PC structures can be divided into Bragg scattering-type and locally resonant-type, both of which are the result of periodicity in composite material structures and of Mie scattering in oscillators [12].

Bragg scattering theory is commonly employed to calculate the BGs of PC structure and result in a high starting frequency over 10 kHz with wide bandwidth [13]. However, to meet the requirement of noise cancellation and vibration suppression, the BGs of the PC structure with both a low starting frequency and large bandwidth is necessary. Therefore, numerous studies showed specific PC structures with low starting frequencies and wide bandwidths by using a new locally resonant mechanism [14]. To investigate the BGs and transmission characteristics of PC structures, finite element method (FEM) is commonly used analysis tool [15,16,17,18]. Lu et al. developed a two-degree-of-freedom locally resonant PC structure with a broad BG under 200 Hz [19]. Zhai et al. proposed a single-scattering structure composed of lead scatterer surrounded by spiral elastic beams which BG was in the range of 50–200 Hz [20]. Li et al. utilized the multiple-scatter theory method to study the BGs of a PC structure and introduce a physical method into the variation of the starting frequency by an analytical method [21]. Taking the full vector character into account, the multiple-scatter theory was extended to out-of-plane propagating elastic beams in two-dimensional (2D) PC structures by Mei et al. [22]. Sainidou et al. calculated the frequency band structure of an infinite PC structure, which consists of a stack of identical slices parallel to a given surface [23]. Liu et al. developed a wavelet-based FEM to calculate BGs and the corresponding transmission characteristics of 2D PC structures [24]. He also presented an array of periodic coating on a thin plate, which were investigated by FEM simulations and experiments [25]. Furthermore, some researchers proposed a generalized structural optimization scheme to optimize 2D PC structures [26,27,28].

Recently, several multiple-scatter PC structures [29] have been presented to design effective tunable acoustic filters in the low frequency range. Romero-Garcia et al. theoretically and experimentally analyzed a 2D multi-resonant acoustic scatterers and found that it was a locally multi-resonant acoustic meta-material (LMRAM) with the strong dispersion [30]. Using FEM simulations, Zhang et al. presented a membrane-type acoustic meta-material embedded with different masses at adjacent unit cells to increase the transmission loss at the low frequency range [31]. However, the regular pattern of arrangement for multiple-scatter PC structure has not been analyzed.

Based on the above analysis, BGs and transmission characteristics of a 2D wrap-around multi-oscillator/scatterer PC structure is proposed and further analyzed using FEM method, especially for the regular pattern of arrangement of the proposed PC structures, such as the 2 × 2, 3 × 3 and 4 × 4 scatterers, the number of connection beams, the size ratio of 2 × 2 scatterers. Several design rules of this type of multiple-scatter PC structure are drawn, which might provide a solid support on the design of such type of acoustical functional materials.

## 2. The Analysis of Single-Scattering Prototype

**Figure 1:**
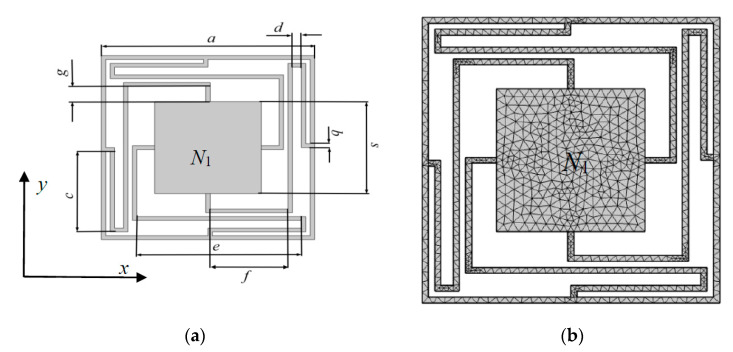
(**a**) The sketch of the phononic crystal (PC) structure; (**b**) The finite element method (FEM) meshes of the proposed PC structure.

Before numerical experiment of the multiple-scatter PC structure, a prototype of 1 × 1 scatterer model base on locally resonant mechanism is proposed. The 1 × 1 scatterer PC structure model is a square lattice unit cell (denoted by N_1_) consists of an oscillator/scatterer surround by four elastic beams. The diagrammatic sketch and FEM mesh diagram are shown in Figure 1, and the corresponding geometry are as follows: *a* is lattice constant of the unit cell, *b* is unique thickness of the elastic beams, *s* is the side length of square scatterer, and *d*, *c*, *e*, *f, g* are the length of each elastic beams, respectively. Furthermore, the thickness of frame is fixed to 0.5 mm.

In the FEM simulations, the geometric parameters of *N*_1_ are: *a* = 24 mm, *b* = 0.5 mm, *c* = 10.5 mm, *d* = 1 mm, *e* = 18.5 mm, *f* = 8.75 mm, *g* = 2 mm, *s* = 12 mm. The material of elastic beams and frame is PA6, and scatterer is piezoelectric ceramic. The mechanical parameters of the two materials are showed in Table 1.

To investigate BGs, the physical governing field equations of elastic wave propagation in the 2D *x*-*y* plane as follows

(1) $$-\rho \left(r\right)\omega^{2}\;u_{i}\;\left(r\right)\;=\nabla \cdot \left[\mu \left(r\right)\nabla u_{i}\left(r\right)\right]+\nabla \cdot \left[\mu \left(r\right)\frac{\partial }{\partial x_{i}}u\left(r\right)\right]+\frac{\partial }{\partial x_{i}}\;\left[\lambda \left(r\right)\nabla \cdot u\left(r\right)\right]\;\;\left(i=x,\;y\right)$$

where the material parameters in above equation are: *ρ*(***r***) is the mass density, ***r*** *= (x, y)* denotes the position, $\nabla =\left(\frac{\partial }{\partial x},\;\frac{\partial }{\partial y}\right)$ is the two-dimensional nabla operator, *ω* is the circular frequency, *μ*(***r***) and *λ*(***r***) are the space-dependent Lame coefficients, and *u_i_*(***r***) (*i = x, y*) represent the two components of the elastic displacement vector ***u***(***r***). On account of the Bloch theorem, the infinite systems concurrently exhibit periodicity along the *x*- and *y*-directions. Therefore, only a unit cell of the PC structure will be calculated with two periodically conditions set on the two opposite boundaries. The periodic conditions on two opposite of the unit cell is represented by

(2) $$\psi \;\left(r+a\right)=e^{ik\cdot a}\psi \left(r\right)$$

where ***ψ*** is phase shift, *r* is a variable situated at the boundaries, ***a*** is the lattice periodical vector, and ***k*** is the wave vector. A Bloch wave vector ***k*** = (*k_x_, k_y_*) related wave on the boundaries is defined by Bloch periodic boundary conditions. The wave vector ***k*** = (*k_x_, k_y_*) on the first Brillouin curves build a dispersion curves for the propagation direction. For the X direction, the BG properties and eigenmodes of the PC structure are deduced by scanning along the irreducible Brillouin zone Г-Х-M-Г, the corresponding wave vector is denoted by *k_._*

**Figure 2:**
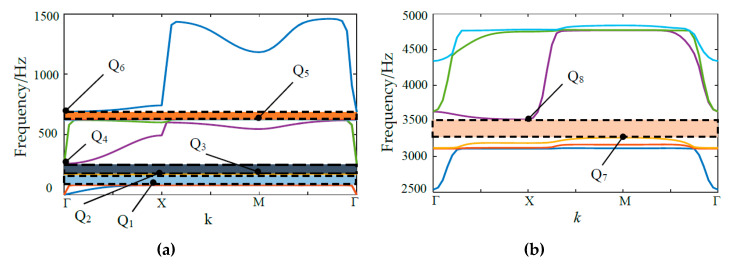
(**a**) The diagram of the first three band gaps (BGs); (**b**) The diagram of the fourth BG.

By utilizing commercial FEM software COMSOL (Comsol Multiphysics 5.4, COMSOL Inc., Stockholm, Sweden.), the characteristic of band structure is calculated and shown in Figure 2, twenty frequency bands are obtained to generate four clear BGs. The first four BGs are within [80.1, 167.1] Hz, [171.8, 257.5] Hz, [632.9, 691.7] Hz, and [3272.6, 3524.2] Hz, respectively. Q_1_, Q_2_, Q_3_, Q_4_, Q_5_, Q_6_, Q_7_ and Q_8_ are the boundary points of all the energy bands.

**Figure 3:**
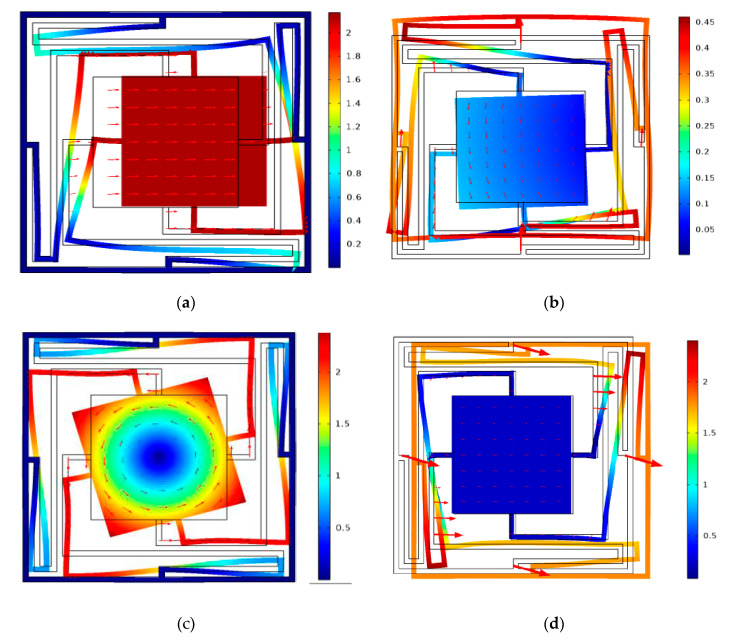

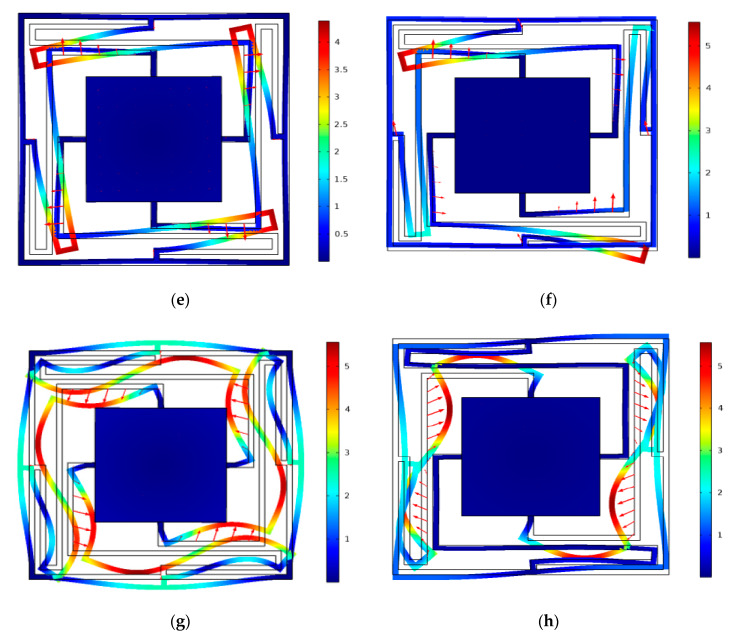
The vibration modes and displacement vector fields of the corresponding point in BGs of the proposed PC structure (**a**–**h**).

The displacement mode shapes of N_1_ in associate with Q_1_, Q_2_, Q_3_, Q_4_, Q_5_, Q_6_, Q_7_ and Q_8_ are shown in Figure 3. Through the analysis of calculation data, the wave vector *k* of each boundary points from Q_1_ to Q_8_ are 0.4167, 0.5, 2, 3, 2, 3, 2, 1 respectively. From Figure 3, the vibration process of the modes severed as a mass-spring system, in which the scatterer plays the role of masses, and the elastic beams and frame work as springs. Mode Q_1_ (80.1 Hz) shows that vibration response is concentrated to the scatterer and its adjacent elastic beams, whereas the frame remains almost stationary. Therefore, it results in a translational resonance mode of the mass-spring system. For Mode Q_3_ (171.8 Hz), the scatterer and elastic beams are found to exhibit torsion resonance mod, whereas the frame is hard to move. For modes Q_2_ and Q_4_, they are obviously that the vibration centralizes in elastic beams and the frame at the frequency of 167.1 Hz and 257.5 Hz, respectively. On the other word, the scatterer is like the rigid boundary and barely moves, which lead to the elastic wave in the unit cell has complex multiple elastic scattering. For modes Q_5_ and Q_6_, the vibration concentrates in elastic beams at 632.9 Hz and 691.7 Hz, and the frame and scatterer have obviously unchanged. Finally, in the higher frequency range, only the frame and elastic beams presented violent vibration, which lead to the widest BG (251.6 Hz) at the modes Q_7_ and Q_8_.

According to the advantages of low frequency and simplicity of the proposed wrap-around PC, the regular pattern of multiple-scatter/ oscillator PC structures are investigated.

## 3. Comparison of 2D Wrap-Around Multiple-Scatter PC Structure

**Figure 4:**
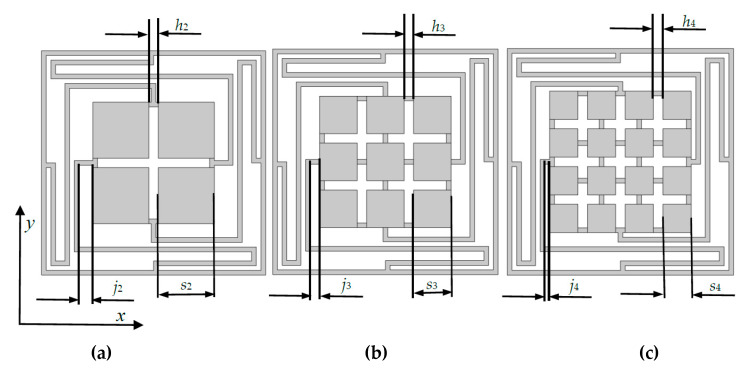
Three designed models for the multiple-scatter PC structures (**a**–**c**).

In this section, the basic 1 × 1 2D multiple-scatter PC structure are designed to 2 × 2, 3 × 3 and 4 × 4 models for the comparison with BGs and transmission characteristics. We keep the total area of central scatterers and the thickness of elastic beams connection between each scatterer to be 12 × 12 mm^2^, and 0.5 m, respectively. In such conditions, the diagram of three models are shown in Figure 4, which called N_2_, N_3_ and N_4_, respectively.

**Figure 5:**
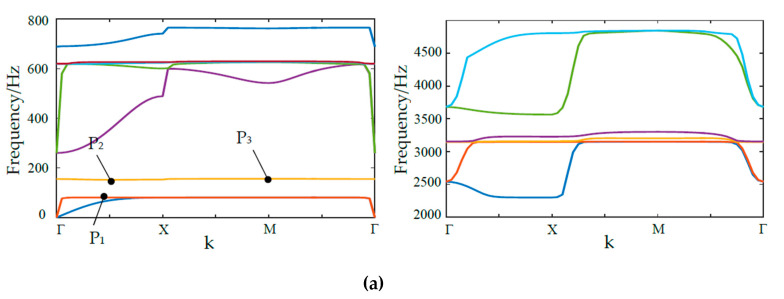

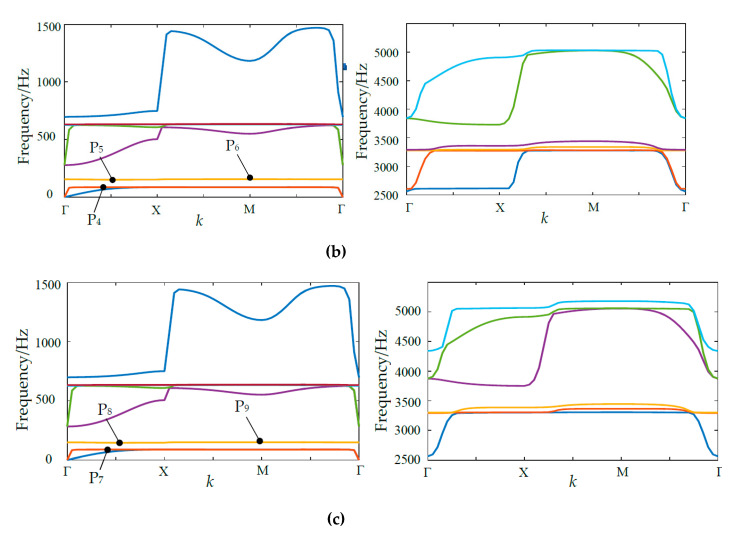
(**a**) Band structure of the PC with N_2_. (**b**) Band structure of the PC with N_3_. (**c**) Band structure of the PC with N_4_.

The parameters of three multiple-scatter PC structures, as shown in Figure 4 are: *h*_2_ = *h*_3_ = *h*_4_ = 1 mm, *s*_2_ = 6 mm, *s*_3_ = 4 mm, *s*_4_ = 3 mm, *j*_2_ = 1.5 mm, *j*_3_ = 1 mm, *j*_4_ = 0.5 mm. Various number of oscillators obstruct the conduction of vibration are analyzed using FEM. The band structures of PC of N_2_, N_3_ and N_4_ are shown in Figure 5. Figure 5a shows the four BGs of N_2_ (within [80.9, 151.9] Hz, [156.2, 260.3] Hz, [630.2, 688.9] Hz, and [3301.2, 3564.6] Hz). Figure 5b shows the four BGs of the PC for N_3_, which are ranged from 86.4 Hz to 151.4 Hz, 156.1 Hz to 277.7 Hz, 635.3 Hz to 696 Hz, and 3439.2 Hz to 3728.6 Hz, respectively. From Figure 5c, the band structure of N_4_ has four BGs (from 87.4 Hz to 144.4 Hz, 149 Hz to 281.7 Hz, 636.6 Hz to 697.8 Hz and 3445.7 Hz to 3751.7 Hz). 

**Figure 6:**
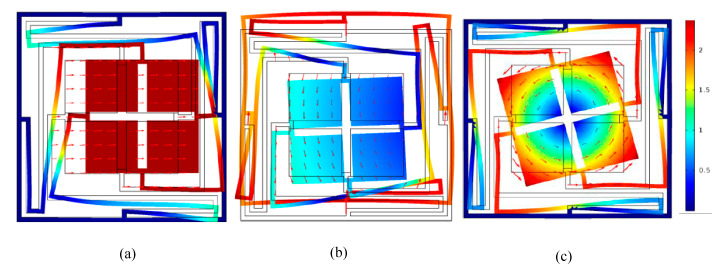

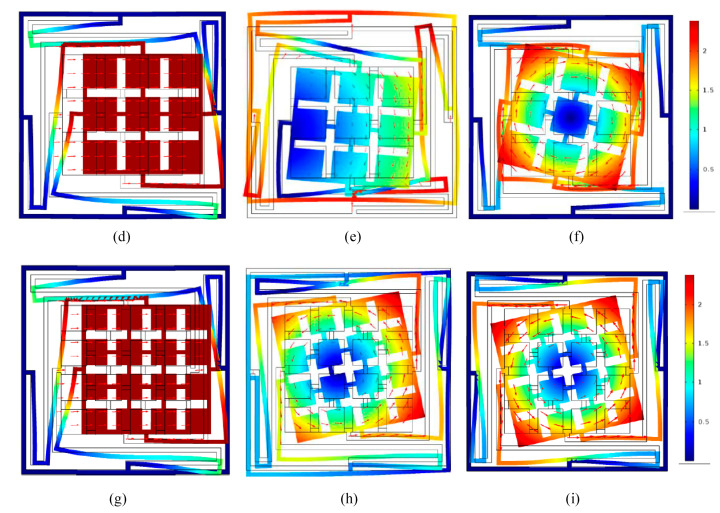
The vibration modes and displacement vector fields of the corresponding point in BGs of the PCs structure (**a**–**i**).

To figure out the physical mechanism for the variation of the low frequency BGs for the different wrap-around multiple-scatter PC structures, the associated edge modes at the edges of first BG boundaries and second BG lower edges are calculated. As shown in Figure 6, it can be found that the edge modes (upper edges and second BG lower edges) of the first BG are changed by comparing with Figure 3. 

**Figure 7:**
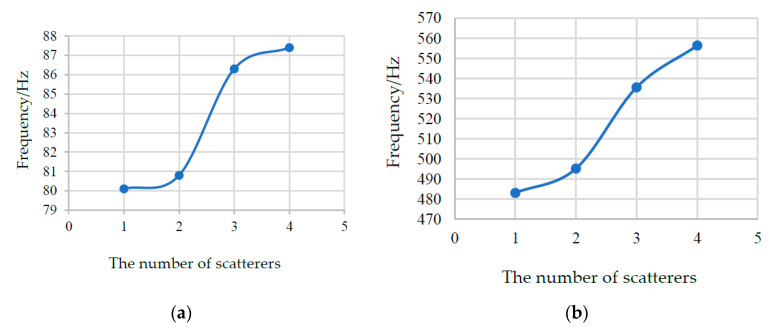
The regular pattern of the starting frequency and the total bandwidth. (**a**) the variation rule of the starting frequency; (**b**) the variation rule of the total bandwidth (four BGs).

Figure 7 shows that the regular pattern of the starting frequency and the total bandwidth. It notes that the number (1, 2, 3, 4) in horizontal coordinate represent N_1_, N_2_, N_3_, and N_4_ PC structures, respectively. By observing Figure 7a, we find that the starting frequency increases slightly with the number of scatterers raises. From Figure 7b, it is clearly that the total bandwidth (four BGs) increase with the number of scatterers grow.

To obtain the transmission spectra, a finite system is obliged to be defined. The structure is finite in *x*-direction is considered that contains five unit cells. Contrary to the *x*-direction, the *y*-direction still utilize the periodic boundary conditions. The plane waves with single frequency, provided by designated acceleration are incident from the left side of finite array and diffused along *x*-direction, the equation of transmission can be defined by:

(3) $${T=10\;\times \;\log}_{10}\frac{v_{in}}{v_{out}}$$

where the variables *v_in_* and *v_out_* in above equation are the value of transmitted and incident displacement, respectively. 

**Figure 8:**
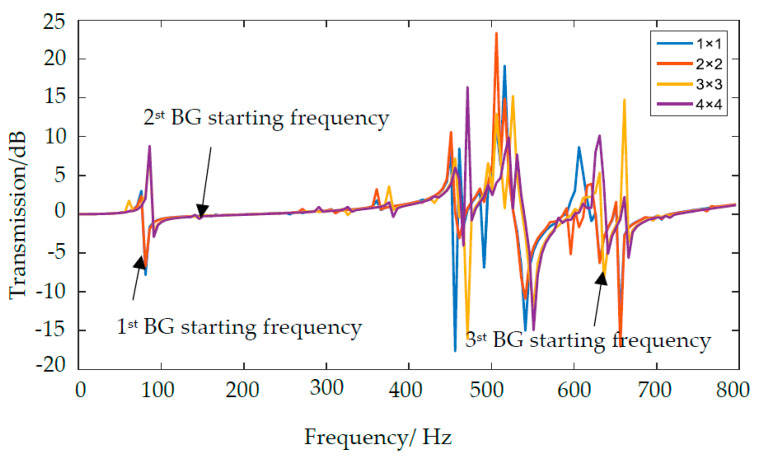
The transmission spectra of N_1_, N_2_, N_3_, and N_4_.

By changing excitation value of the incident displacement, the transmission spectra are obtained and drawn in Figure 8. From Figure 8, the transmission curve exists attenuation in the first, second and third BG range of the N_1_, N_2_, N_3_ and N_4_ PC, and the blue, orange, yellow, purple line denote transmission spectra of N_1_, N_2_, N_3_, and N_4_, respectively. It is obviously that the transmittance of 2 × 2 model (N_2_) is smaller than the others within the first, second and third BG ranges of four PCs, which verify the vibration insulation effect of 2 × 2 model is quality. 

Based on the transmission spectra and the characteristics diagram of band structure, several conclusions are drawn: with the number of oscillators increase, the mass and volume of each scatterers reduce which lead to the natural frequency of oscillators increase. Due to the number of scatterers increase, the structure has abundant vibration modes, which might bring about the negative effect for the coupling of each oscillator; more oscillators can open a much wider total bandwidth near the scatterers. N_2_ equips with the relative best vibration insulation effects.

To sum up, considering the starting frequency and vibration insulation effects, N_2_ (2 × 2 model) is the relative optimal multiple-scatter structure. Therefore, we further analyze the regular of size ratio of N_2_ to influence BGs in Section 4.

## 4. The Regular Pattern of Size Ratio on the 2 × 2 Model

**Figure 9:**
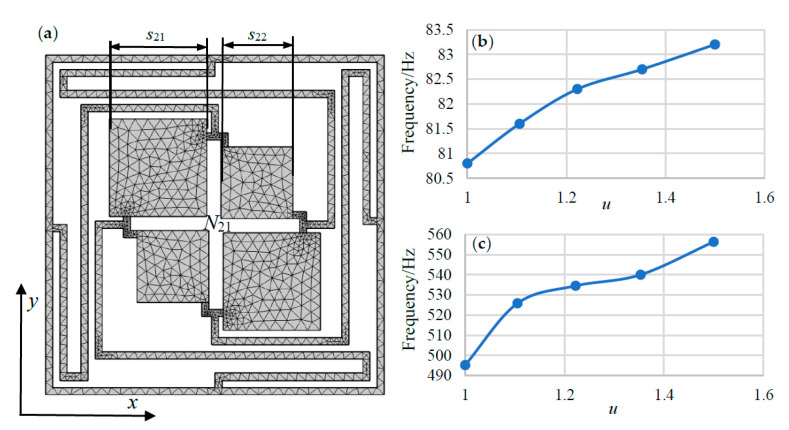
N_21_ and the variation regulars of the starting frequency and the total bandwidth: (**a**) N_21_ multiple-scatter PC structure. (**b**) the starting frequency. (**c**) the total bandwidth.

As shown in Figure 9a (N_21_), to investigate the influence of the size ratio of two neighbor scatters/oscillators, we remain the total area unchanged, and take the four symmetrical oscillators retain the ratio *u*, which is represented by:

(4) $$u={\left(\frac{s_{21}}{s_{22}}\right)}^{2}$$

where *s*_21_ and *s*_22_ are the side lengths of neighbor unit cells.

Because the four oscillators are changed with size proportion which keep central symmetry (the first and fourth oscillators are adopted as the bigger side), N_21_ is not the perfectly symmetrical model like N_2_. The variation regular of the starting frequency and the total bandwidth for N_21_ are shown in Figure 9b,c, respectively. Due to the space limitation of N_21_, the ratio *u* is set to 3:2, 23:17, 11:9, 21:19 and 1:1, respectively. As illustrated in Figure 9b,c, the tendency of starting frequency and total bandwidth are increase with a large *u*. More specifically, the vibration effect is influence by the area disparity of scatterers which abate the vibration effect of oscillators.

Though the above analysis, the conclusion is obtained: the increase of scatterers area disparity will leads to the different coupling between the large and small one connect by elastic beams and further influence the variation of BGs; the relative best size proportion of the scatterers is obtained which is 21:19, which has the lower starting frequency and wider bandwidth.

## 5. Research on the Number of Connection Beams

**Figure 10:**
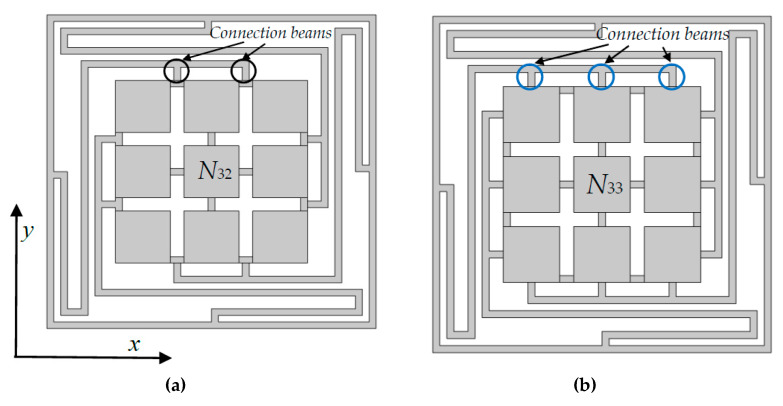
The diagrams of the connection beams on 3×3 model. (**a**) *O*c = 2; (**b**) *O*c = 3.

The number of connection beams are generally influencing the BGs. Models (we only put the 3 × 3 models for exhibition) are given in Figure 10. During the investigation, we discover a new law that the number of connection beams between scatterers and the elastic beams on periphery. Take one side of the scatters/oscillators for example, as shown in Figure 4b, Figure 10a,b, the number of connection beams *O*_c_ = 1, 2 and 3, respectively. Obviously, *O*_c_ is depend on the numbers of oscillators. In 2 × 2, 3 × 3, 4 × 4 models, *O*_c_ are changed from 1 to 2, 1 to 3, 1 to 4, respectively.

**Figure 11:**
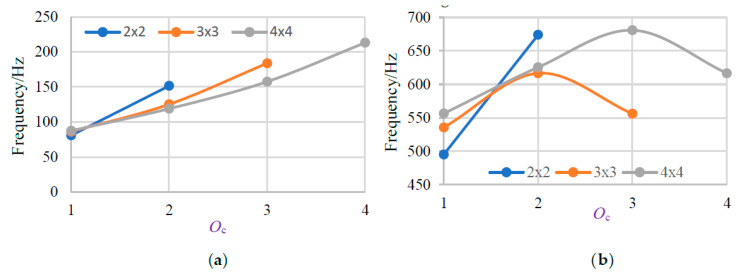
(**a**) The change rule of the starting frequency in associate with *O*_c_. (**b**) The change rule of the total bandwidth in associate with *O*_c_.

The regular patterns or change rules of the starting frequency and the total bandwidth in associate with the number of connection beams *O*_c_ are shown in Figure 11. It is obviously that the starting frequency of all the three models and the total bandwidth of 2 × 2 model grow with a large *O*_c_. At the same time, the total bandwidth first increases then decreases with the increasing of *O*_c_ for 3 × 3 and 4 × 4 models.

**Figure 12:**
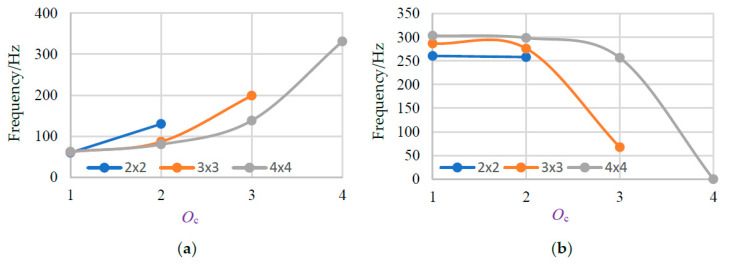
(**a**) The change rule of bandwidth for the third BGs with different *O*_c_. (**b**) The change rule of bandwidth for the fourth BGs with different *O*_c_.

To further investigate the change of total bandwidth, we draw the change regular of bandwidth for the third and the fourth BGs, as shown in Figure 12. It can be used for interpreting changes of total bandwidth.

From Figure 12a, the bandwidth of the third BG is increased with a large *O*_c_, but the bandwidth of the fourth BG is decreased accordingly. By scrutinizing the data in previous regular, the third BG increase which in previous typically do not vary, and the changes of the fourth BG is relatively large which lead to the bandwidth plunge to 0 Hz and make total bandwidth lessen. It is considered that two connection beams have the best performance on BGs of the present multiple-scatter PC structure.

## 6. Conclusions

In this paper, the BGs and transmission characteristics of a 2D wrap-around multiple-scatter PC structure with periodic arrangement of scatters/oscillators are investigated using commercial FEM software COMSOL. The wrap-around structure provides locally resonators for obtaining four BGs in different frequency ranges. Combined with locally resonance mechanism, the first three complete BGs are found in low frequency ranges and the fourth BG in the high frequency range. 1 × 1 model, 2 × 2, 3 × 3 and 4 × 4 scatters/oscillators models are designed and compared with the basic 1 × 1 scatter structure, and the change rule is obtained that the starting frequency and the total bandwidth of 4 BGs are increase as the number of scatterers increase. The relative proportions of scatterers are studied and the research results verify a significant advantage on lower starting frequency and wider bandwidth of four BGs. By increasing the number of connections of scatterers with elastic beams, the starting frequency present increase trend and the width of 4 BGs exhibit parabolic trend (parabola going downwards). The present investigation of multiple-scatter PC structures will provide a support on the future design of multiple-scatter PC structures severed as acoustical functional materials. Further works will be focused on the study of piezoelectric properties of beams not scatterers and using maturity 3D printer technological to test the sound insulation with better performance. 

## Figures and Tables

**Table 1:** The mechanical parameters of the two materials.

Materials	Density (*ρ*) (Kg/m)	Young’s Modules(E) Pa	Poisson’s Ratio (*ν*)
PA6	1180	2.32 × 10^9^	0.39
Piezoelectric Ceramic	7500	76.5 × 10^9^	0.32
